# Drivers of Epilithic Biofilms in Greenland Streams: The Role of Nutrients, Temperature and Catchment Slope Across a Climate Gradient

**DOI:** 10.1111/1758-2229.70074

**Published:** 2025-03-12

**Authors:** Sanne M. Moedt, Kirsten S. Christoffersen, Andreas Westergaard‐Nielsen, Kenneth T. Martinsen, Ada Pastor, Niels Jákup Korsgaard, Tenna Riis

**Affiliations:** ^1^ Department of Biology University of Copenhagen Copenhagen Denmark; ^2^ Arctic Biology, University Centre in Svalbard Norway; ^3^ Department of Geosciences and Natural Resource Management University of Copenhagen Copenhagen Denmark; ^4^ Institute of Aquatic Ecology, University of Girona Girona Spain; ^5^ Geological Survey of Denmark and Greenland Copenhagen Denmark; ^6^ Department of Biology Arctic Research Center, Aarhus University Aarhus Denmark

**Keywords:** aquatic microbiology, biofilm biology, microbial communities

## Abstract

The Arctic is warming faster than the global average, making it critical to understand how this affects ecological structure and function in streams, which are key Arctic ecosystems. Microbial biofilms are crucial for primary production and decomposition in Arctic streams and support higher trophic levels. However, comprehensive studies across Arctic regions, and in particular within Greenland, are scarce. This study analysed total biomass, autotrophic biomass (chlorophyll *a*), and the general structure of major autotrophic groups in stream epilithic biofilms across Greenland's subarctic, Low Arctic, and High Arctic regions. Our aim was to identify primary environmental drivers of biofilm across these climate regions. We observed large environmental variation differences in biofilm chlorophyll *a* concentrations and total biomass across the regions. Cyanobacteria, diatoms, and green algae were present in all regions, with cyanobacteria dominating High Arctic streams. Phosphate and water temperature primarily drove autotrophic biofilm abundance measured as chlorophyll *a* concentration, while catchment slope and nitrate concentrations influenced total biofilm biomass, with relationships varying by region. Our results suggest increased biofilm accumulation in Greenland streams under projected climate warming, which likely will alter trophic food webs and biogeochemical cycling, with region‐specific responses expected.

## Graphical abstract

Microbial biofilms are crucial components in Arctic streams, and understanding how climate warming affects them is critical. Across three regions in Greenland, we found large differences in biofilm structure and their drivers. Our results suggest increased but region‐specific biofilm accumulation in Greenland streams under a warmer climate, which may alter trophic structure and biogeochemical cycling significantly.

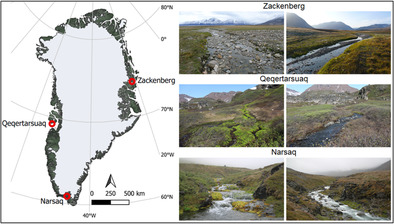

## 1 Introduction

Stream ecosystems in the Arctic are currently subjected to multiple environmental changes as a result of climate change (Vonk et al. 2015; Wrona et al. 2016). Changes such as increased temperatures and altered precipitation patterns directly or indirectly affect the physical–chemical conditions in streams (Docherty, Riis, Milner, et al. 2018; Huryn 2020), which will lead to shifts in ecosystem functioning on a circumpolar scale. Especially in nutrient‐poor streams, ecosystem primary production is dominated by autotrophic microbial biofilms, that is, attached to a substrate (Battin et al. 2016; Busi et al. 2022; Quesada et al. 2008). Additionally, biofilms as such play an important role in other ecological processes, such as organic matter degradation (Kohler et al. 2022; Sudlow et al. 2023) and nutrient cycling (Busi et al. 2022). They also form the basis of the aquatic food web (Battin et al. 2016), especially in nutrient‐poor freshwaters such as those in the Arctic where terrestrial plant detrital inputs are scarce (Vander Zanden and Vadeboncoeur 2020). Therefore, understanding how climate and other environmental factors shape Arctic stream biofilms is critical to predict future changes in ecosystem functioning due to climate change.

Epilithic stream biofilms consist of a complex assemblage of autotrophic and heterotrophic microbes, embedded in a polysaccharide matrix attached to submerged stones and rocks (Battin et al. 2016) and with specific environmental requirements for growth. Autotrophs in biofilm assemblages consist primarily of diatoms, green algae, and cyanobacteria (Sudlow et al. 2023), whereas heterotrophs are predominated by bacteria, fungi, protozoans and meiobenthos (Battin et al. 2016; Risse‐Buhl et al. 2012; Weitere et al. 2018). These two microbial components complement each other, where autotrophs provide heterotrophs with organic compounds (e.g., carbohydrates and amino acids), while heterotrophs produce carbon dioxide which can be taken up by autotrophs (Battin et al. 2016, 2003). This internal biofilm carbon cycling can be rather self‐sufficient as long as there is enough light and inorganic nutrients (Romaní et al. 2004).

Previous studies show that epilithic biofilms in Arctic streams are either limited by nitrogen or co‐limited by nitrogen and phosphorus (Hauptmann and Myrstener 2023; Myrstener et al. 2018; Pastor et al. 2020). For instance, during a nutrient addition experiment, Hauptmann and Myrstener (2023) have demonstrated that added nitrogen and phosphorus promoted biofilm biomass accumulation. Physical attributes of catchments, such as size, slope, and vegetation cover, can be other important but indirect factors driving biofilm biomass, as they have been shown to influence nutrient availability in streams (Harms et al. 2016; Riis et al. 2023). For example, Connolly et al. (2018) found a positive correlation between watershed slope and summer nitrate concentrations across the Arctic.

Greenland constitutes an important component within the circumpolar Arctic as it deviates from most other Arctic regions due to its size and its permanent ice sheet, which strongly impacts the local climate and ecosystem functioning (DeBeer et al. 2020; Heindel et al. 2015). Additionally, Greenland is geographically isolated, which causes dispersal limitations for certain organisms, such as insects and fish (Lento et al. 2022). Furthermore, Greenland encompasses three distinct climate zones, that is, the subarctic, Low Arctic and High Arctic (Lento et al. 2019). These zones differ in annual mean temperature, precipitation patterns, light climate, and permafrost extent. Understanding stream biofilm ecology along this large climatic gradient in Greenland can enhance our global understanding of biofilm diversity across the broader Arctic.

In this context, studies focusing on the ecology and composition of biofilm assemblages and those covering large geographical areas in the Arctic are scarce (but see Heikkinen et al. 2023; Kahlert et al. 2022; Puts et al. 2022). In particular, it is notable that comprehensive studies of stream biofilms in Greenland are few and limited to specific study areas, thus hampering a broader understanding of biofilm dynamics and environmental interactions across different climatic regions in the whole Arctic.

The goal of this study was twofold: (i) to determine biomass and relative abundance of major autotrophic groups of epilithic biofilms and (ii) to identify the main environmental drivers across three climate regions in small, clear‐water Greenland streams. To do that, we obtained biofilm samples and measured environmental variables across a large environmental gradient in Greenland, encompassing three climate regions: subarctic, Low Arctic, and High Arctic. We hypothesised that (1) climate (temperature and precipitation) will drive autotrophic and total (autotrophic and heterotrophic) biofilm biomass; (2) catchment area, slope and vegetation cover will positively affect biofilm biomass due to increased allochthonous organic matter inputs into the streams and the associated heterotrophic microbial production (3) and availability of inorganic nutrients (nitrogen and phosphorus) in the water column will influence the biomass of primary producers, thereby affecting total biofilm biomass due to changes in primary production and biomass accumulation. The understanding of stream microbial biofilms along Greenland's environmental gradient offers invaluable insights into the role of stream biofilms in the Arctic, thereby enriching our comprehension of their ecological significance.

## 2 Methods

### 2.1 Study Sites

**FIGURE 1:**
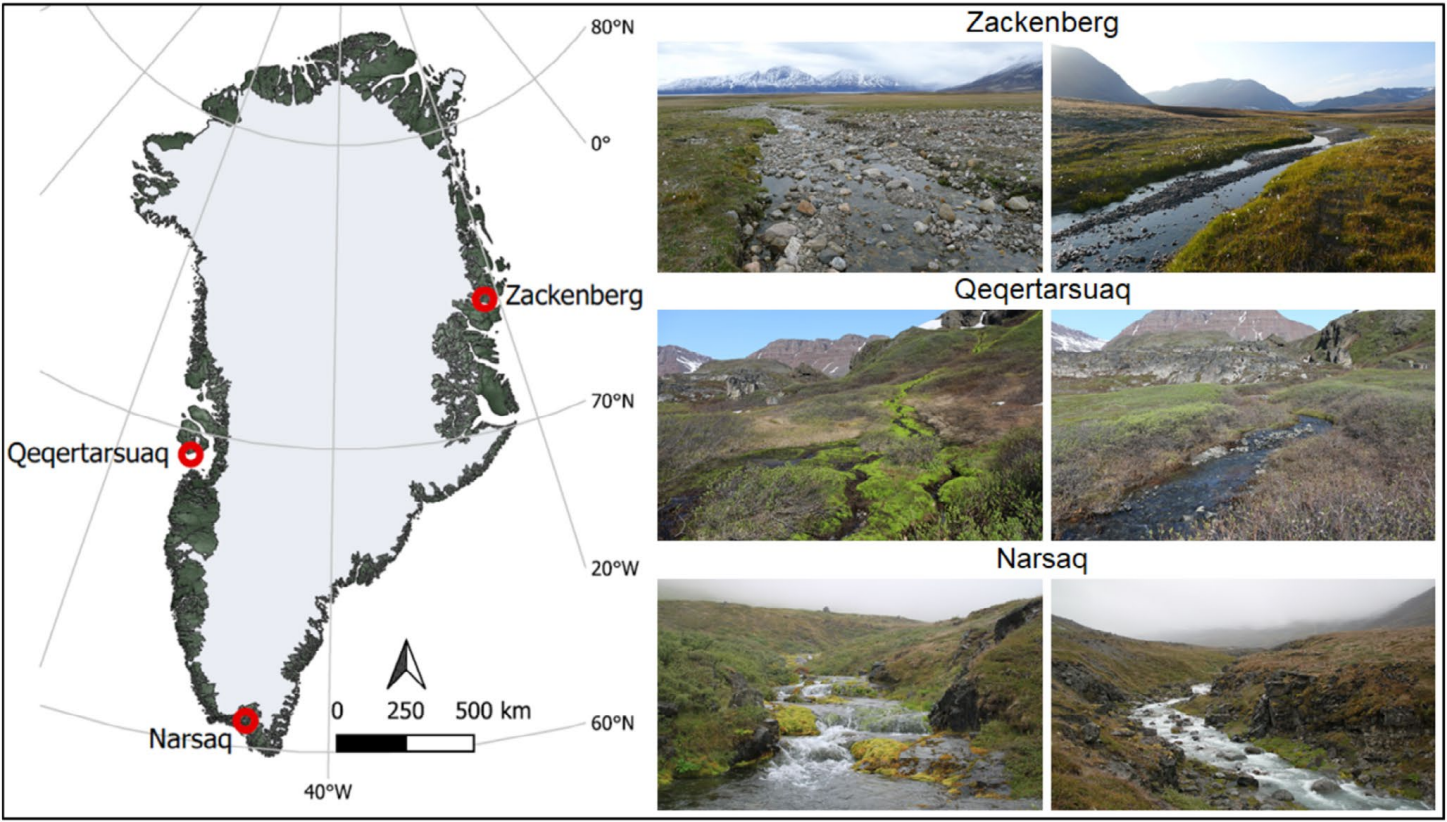
Map of Greenland with the studied regions (Zackenberg, Qeqertarsuaq and Narsaq) and photos of stream sites in each region. The map was created using QGIS version 3.4. Photo credit: Zackenberg by A. Pastor, and Qeqertarsuaq and Narsaq by S. M. Moedt.

We studied 28 streams in three different climate regions in Greenland (Figure 1). These were as follows: Zackenberg (74°29′44″ N 20°45′59″ W) in the High Arctic region, Qeqertarsuaq (69°14′50″ N 53°32′00″ W) in the Low Arctic region, and Narsaq (60°54′44″ N 46°02′55″ W) in the subarctic region. The duration of polar night differs markedly from north to south, with 89 days in Zackenberg and 44 days in Qeqertarsuaq, while there is no polar night in Narsaq. The catchment area was dominated by tundra vegetation in Zackenberg and Qeqertarsuaq (Callaghan et al. 2011; Elberling et al. 2008), whereas in Narsaq the vegetation varied from grassland in the lowland riparian areas to tundra vegetation at the high altitudes in the catchment (Rose‐Hansen et al. 1977; Westergaard‐Nielsen et al. 2015). All regions are mountainous with several streams originating from glaciers. Qeqertarsuaq has geothermal springs, which feed into some of the streams in the area (stream SE, SK and SS). Water chemistry and biofilm samples were collected from 14 streams in Zackenberg between the 20th of August and the 3rd of September 2017 (Pastor et al. 2019), from six streams in Qeqertarsuaq between the 18th and 25th of September 2018 (Pastor et al. 2021), and from eight streams in Narsaq between the 4th and 6th of August 2022 (Table S1). Additional measurements for major autotrophic group relative abundance in Qeqertarsuaq and Zackenberg were conducted in July and August 2023, respectively. Several of these sites were not identical but should still be representative for the respective regions.

### 2.2 Meteorological Information

Data on local air temperature and precipitation during the 10 years prior to biofilm sampling in Zackenberg and Qeqertarsuaq was obtained from the Greenland Ecosystem Monitoring database (measured at 2 m above the ground; https://data.g‐e‐m.dk/) and data from Narsaq (Narsarsuaq, 40 km away) was obtained through the Danish Meteorological Institute (DMI, http://research.dmi.dk/publications/other‐publications/reports/). We calculated annual and summer (July and August) mean air temperature and accumulated precipitation. For Qeqertarsuaq, precipitation data were only available from 2010 onwards, and for Zackenberg 2014, precipitation data were incomplete and consequently removed from the dataset.

**FIGURE 2:**
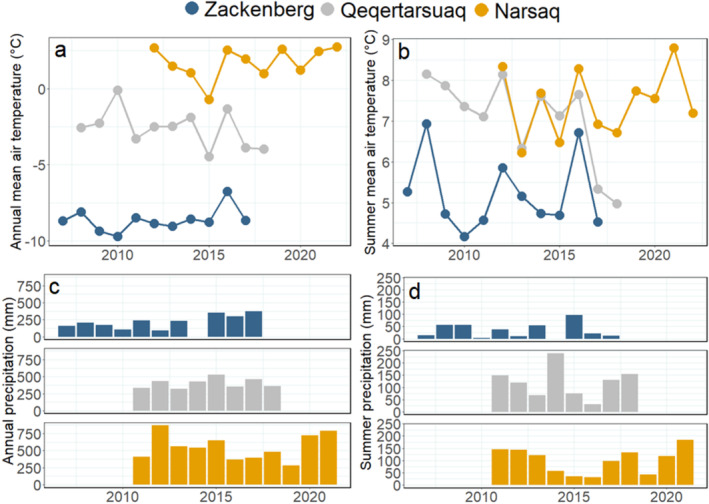
Annual (a) and summer (b) mean air temperature (°C) during the 10 years prior to sampling and during the sampling year in Zackenberg (blue), Qeqertarsuaq (grey), and Narsaq (orange). Precipitation data for Qeqertarsuaq were only available from 2010 onwards.

During the 10 years prior to biofilm sampling, Zackenberg had an annual mean air temperature of −6°C to −10°C (Figure 2a) and a summer mean temperature of 4°C–7°C (Figure 2b). Annual precipitation was between 100 and 400 mm (Figure 2c) and summer precipitation around 10–100 mm (Figure 2d). In the Qeqertarsuaq region, the annual mean air temperature was between 0°C and −5°C, while summer temperatures were between 5°C and 8°C. The typical annual precipitation in the Qeqertarsuaq area is around 400 mm (Zhang et al. 2019). Finally, Narsaq had the highest annual and summer mean air temperatures (1°C–2°C and 6°C–9°C, respectively) and annual and summer precipitation (250–800 and 25–200 mm, respectively) in the 10‐year period.

**FIGURE 3:**
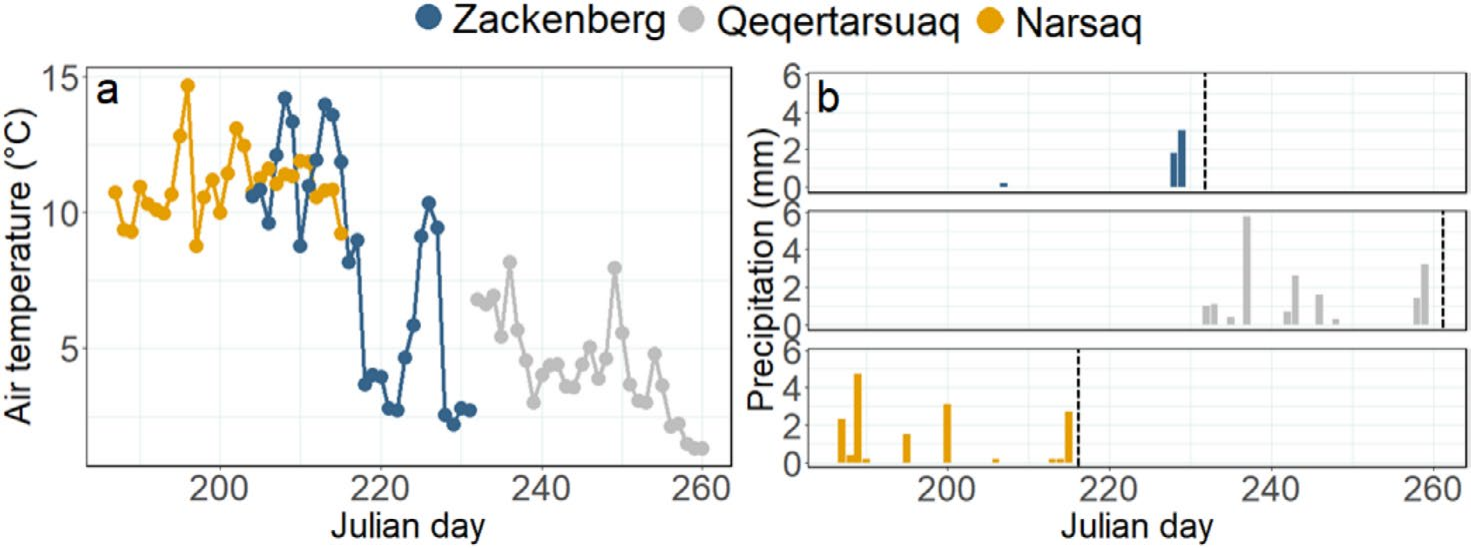
Daily mean air temperature (°C) (a) and precipitation (mm) (b) during the 4 weeks prior to the start of sampling in Zackenberg (blue), Qeqertarsuaq (grey), and Narsaq (orange). The vertical dashed line indicates the onset of sampling within each region.

During the 4 weeks prior to the start of sampling, the mean air temperature at Zackenberg was 8°C, in Qeqertarsuaq 4°C, and in Narsaq 11°C (Figure 3a). During these 4 weeks, total precipitation in Zackenberg was 5 mm, in Qeqertarsuaq 18 mm, and in Narsaq 16 mm (Figure 3b).

### 2.3 Catchment Topography and Vegetation

We determined catchment characteristics, including total area, mean elevation, mean slope, and normalised difference vegetation index (NDVI), as a proxy for vegetation (Myers‐Smith et al. 2020), for each stream catchment. The tools used differed slightly between the three regions.

At each of the three sites, we used 2 m digital elevation models (DEM; see Data S1) to delineate the catchments to each water sample point and characterise slope and aspect within the catchments. The DEMs were preprocessed using least‐cost breaching (Lindsay and Dhun 2015) and depression filling (Wang and Liu 2006). Flow directions were determined using the D8 algorithm (O'Callaghan and Mark 1984), which was subsequently used to define the catchment boundaries and flow accumulation.

In Zackenberg and Narsaq, the workflow was performed in a QGIS environment, and for Qeqertarsuaq, we used ESRIs ArcGIS Pro. We processed spatial NDVI within each catchment using atmospherically corrected Sentinel‐2, level 2A data, providing bottom of atmosphere surface reflectance. The spatial resolution is 10 m. At all three sites, we used the Sentinel‐2 scenes closest in time to the biofilm sampling. In Zackenberg and Narsaq, the NDVI processing was done with R and GDAL, and in Qeqertarsuaq, we used preprocessed NDVI from the GEM database (www.G‐E‐M.dk).

### 2.4 Physical–Chemical Parameters

Water temperature and conductivity were measured in situ by a YSI Multi Sonde approx. 10 cm below the water surface. Water velocity was measured with a velocity metre (Höntzsch flowmeter) at each site over a known profile at the deepest part of the stream channel. Water samples were collected from the middle of the stream and filtered in the field for nutrient analyses, using 0.2‐μm membrane filters (Supor 200 PES, Pall Corporation, Port Washington, New York, US) and for dissolved major ions using 0.7‐μm pre‐combusted glass fibre filters (GFF, Whatman, UK). We froze these samples at −18°C within 6 h after sampling and kept them frozen until analysis, while samples for alkalinity were kept in the fridge at 5°C. Samples for dissolved Si were analysed on an ICP‐MS (PerkinElmer Instruments, Optima 2000 DV). We analysed NO_3_^−^, NH_4_^+^ and PO_4_^3−^ by using a Lachat QC‐8500 Flow Injection Autoanalyzer (colorimetric analysis; Lachat Instruments, Loveland, Colorado, US; APHA 2005). All NO_3_^−^ concentrations were above the quantification limit, but not all NH_4_^+^ and PO_4_^3−^ concentrations (quantification limit for NO_3_^−^, NH_4_^+^ and PO_4_^3−^ was 0.004, 0.010 and 0.005 mg L^−1^, respectively). In Qeqertarsuaq and Zackenberg, triplicate water samples were collected, from which we calculated the mean, whereas in Narsaq, only one sample per site was collected.

### 2.5 Biofilm

We collected epilithic biofilm samples from three replicate sites along a 50 m long reach in each study stream, starting from where water samples were collected and going upstream. At each site, we collected three or four relatively flat stones, arranged them in a tray (20 × 30 cm) with a measuring scale, and took a photo that was used for later digital determination of the stones' surface area, by means of the free software ImageJ (Rueden et al. 2017). Subsequently, we used a BenthoTorch (bbe Moldaenke, Germany) pigment reader to assess the relative abundance of major biofilm microbial autotrophs (cyanobacteria, diatoms and green algae) by means of pigments on three randomly picked stones in each tray. For Qeqertarsuaq and Zackenberg, this was done during a later sampling campaign in 2023, in streams within the same area as described in Section 2.1. Therefore, site names for these measurements are different. Following this, we removed the biofilm from the light‐facing stone side by brushing it off with a toothbrush and concurrently adding small amounts of stream water until the stone surface was clean. This produced a slurry of 100–200 mL, which was collected in a bottle and stored in the dark until further processing later that same day.

For each biofilm sample, we filtered a small quantity of biofilm slurry onto pre‐combusted GF/F filters (Whatman, UK) in Qeqertarsuaq and Narsaq and onto pre‐combusted GF/C filters (Whatman, UK) in Zackenberg for measurements of chlorophyll *a* (Chl *a*) concentration. Additionally, we filtered a small quantity of slurry onto pre‐combusted GF/F filters to measure ash‐free dry weight (AFDW). All filters were individually wrapped in aluminium foil and stored at −20°C until further analysis. The amounts were then scaled to surface area.

In the laboratory, each Chl *a* filter was thawed, added 96% ethanol and placed in darkness for overnight extraction of pigments. The next day, the extracts were re‐filtered to remove any debris and measured in a spectrophotometer at 665 and 750 nm. Chl *a* concentrations were calculated per cm^−2^ of stone surface (light‐facing side). Filters for AFDW were first dried at 60°C for 48 h, and their dry weight was measured. Next, the filters were placed in a furnace at 450°C for 5 h and weighed again. The difference in weights was then used to calculate AFDW per cm^−2^ of stone surface. We also calculated the autotrophic index (AI) as the ratio of mg AFDW to mg Chl *a* per stone surface area that indicates the relative proportion of heterotrophic organisms or non‐living organic material versus autotrophic community contribution in the biofilm (Steinman et al. 2017).

### 2.6 Statistical Analysis

First, ANOVA's were performed to identify differences and similarities in environmental and catchment variables and biofilm characteristics among the regions. Prior to analysis, residuals were examined to verify the assumptions of ANOVA were met. Second, a principal component analysis (PCA) of environmental and catchment factors (selected using forward selection) was calculated with the *princomp* function (*FactoMineR* package v4.2.3) and visualised using *factoextra* (v4.2.3). Third, Spearman's rank correlations were done to identify significant correlations between biofilm characteristics, physical–chemical data and environmental data. Furthermore, these correlations were used to identify candidate predictors for subsequent multiple regression analyses of biofilm characteristics based on significant correlations. Next, to analyse the relationship between biofilm characteristics and physical–chemical, environmental and catchment variables, we initially built linear mixed models (LMM), with region as a random effect. However, this did not lead to significant models, probably due to contrasting relevant drivers for biofilm across the regions. Therefore, we performed multiple linear regressions for each region separately and selected the best model using forward selection based on the Akaike Information Criterion (AIC). Normality of residuals was tested using a Shapiro–Wilk test, and homogeneity of variances was checked visually. All statistical analyses were performed using R version 4.2.0 (2022‐04‐22) through RStudio 2023.09.1.

## 3 Results

### 3.1 Catchment Characteristics

Catchment areas in Narsaq ranged from 5.16 to 36.13 km^2^ and were on average larger than in Qeqertarsuaq and Zackenberg (0.20–3.89 and 0.34–11.26 km^2^, respectively; *df* = 2; *F* = 67.40; *p* < 0.0001 for both; Table 1). Catchment slope was lowest in Zackenberg (*df* = 2; *F* = 70.69; *p* < 0.0001 for both), where it ranged from 1° to 21°. In Narsaq and Qeqertarsuaq, catchment slope ranged from 17°–25° to 15°–27°, respectively. There was no significant difference in mean NDVI among the regions (ANOVA; *df* = 2; *F* = 2.55; *p* = 0.085), which ranged from 0.03 to 0.37 in Zackenberg, −0.01 to 0.41 in Qeqertarsuaq, and 0.16–0.32 in Narsaq (Table 1).

**TABLE 1:** Catchment and physical–chemical characteristics of the study streams in the three regions: Zackenberg, Qeqertarsuaq, and Narsaq.

	Stream	Catchment area (km^2^)	Catchment slope (°)	Catchment NDVI	Water temperature (°C)	Velocity (m s^−1^)	Conductivity (μS cm^−1^)	NH_4_^+^ (μg N L^−1^)	PO_4_^3−^ (μg P L^−1^)	NO_3_^−^ (μg N L^−1^)	Si (mg L^−1^)	Julian day
Zackenberg	AE	3.84	6	0.04	6.2	0.49	473	38	4	231	n.a.	242
	GA	1.14	10	0.34	3.1	0.00	387	22	3	176	n.a.	239
	GB	1.43	9	0.37	2.7	0.01	237	37	10	13	n.a.	239
	GC	2.45	1	0.33	3.0	0.02	252	46	4	6	n.a.	237
	KO	0.64	15	0.23	8.3	0.07	302	105	5	134	n.a.	236
	KA	0.86	8	0.14	6.4	0.07	362	22	7	161	n.a.	236
	KB	2.82	10	0.28	6.8	0.08	299	29	2	10	n.a.	233
	KC	4.72	2	0.31	6.9	0.07	293	23	2	5	n.a.	232
	LT	0.48	10	0.04	2.9	0.39	566	28	3	24	n.a.	242
	P	11.26	3	0.03	4.2	0.32	162	29	9	8	n.a.	242
	S1	0.34	5	0.27	3.6	0.23	65	31	4	219	n.a.	243
	S2	1.66	21	0.08	4.3	0.45	41	21	2	4	n.a.	243
	S3	4.95	11	0.23	8.4	0.18	80	55	4	20	n.a.	243
	U	4.42	12	0.31	5.2	0.53	381	42	6	18	n.a.	242
Qeqertarsuaq	SA	1.30	15	0.26	0.2	0.001	90	17	9	18	5.49	265
	SB	0.34	17	0.41	0.7	0.04	77	14	8	5	4.74	265
	SE	0.20	27	0.35	4.0	0.37	63	14	16	36	4.67	268
	SK	0.46	18	0.41	n.a.	0.11	102	16	27	95	5.00	261
	SL	3.89	20	0.00	0.8	0.001	56	18	18	44	2.66	261
	SS	0.63	19	0.28	1.4	0.15	63	14	16	51	3.24	265
Narsaq	NR01	5.16	25	0.16	2.6	0.60	39	0	3	27	0.87	218
	NR02	11.45	21	0.24	5.5	n.a.	46	0	6	15	0.78	218
	NR03	14.69	21	0.27	5.8	0.80	52	44	11	95	1.07	216
	NR05	18.58	20	0.32	6.8	0.80	52	8	8	15	1.24	216
	NR06	30.54	19	0.27	7.2	n.a.	52	3	6	11	0.27	216
	NR07	34.56	19	0.31	7.4	0.79	63	10	6	7	0.31	217
	NT04	11.03	17	0.16	9.4	0.80	55	8	4	25	0.16	216
	NT09	36.13	19	0.32	8.8	0.16	51	1	11	6	0.49	218

*Note:* The data are collated from different sampling campaigns (see Section 2 for details). n.a. means not available.

### 3.2 Physical–Chemical Characteristics

Stream water temperature across the sites in Qeqertarsuaq ranged from 0.2°C to 1.4°C and was lower than in Narsaq and Zackenberg (2.6°C–9.4°C and 2.7°C–8.4°C, respectively; Table 1; ANOVA; *df* = 2; *F* = 37.37; *p* < 0.0001 for both). The velocity in Narsaq streams ranged from 0.16 to 0.80 m s^−1^ and was higher than in Qeqertarsuaq and Zackenberg (0.001–0.37 and 0.00–0.53 m s^−1^, respectively; *df* = 2; *F* = 45.88; *p* < 0.0001 for both). Zackenberg streams had a higher conductivity (41–473 μS cm^−1^; *df* = 2; *F* = 36.06; *p* < 0.0001 for both) than in Narsaq and Qeqertarsuaq (39–63 and 56–102 μS cm^−1^, respectively). Ammonium concentrations were also higher in the streams in Zackenberg (21–105 μg N L^−1^) than those in Narsaq (< 10–44 μg N L^−1^) and Qeqertarsuaq (14–18 μg N L^−1^; *df* = 2; *F* = 21.41, *p* < 0.0001 and *p* = 0.0001, respectively). Phosphate concentrations were higher in Qeqertarsuaq (8–27 μg P L^−1^; *df* = 2; *F* = 50.00; *p* < 0.0001 for both) than in Narsaq (< 5–11 μg P L^−1^) and Zackenberg (< 5–10 μg P L^−1^). Lastly, nitrate concentrations were higher in Zackenberg than in Narsaq (4–231 and 6–95 μg N L^−1^, respectively; *df* = 2; *F* = 4.28; *p* = 0.017). In Qeqertarsuaq, nitrate concentrations ranged from 5 to 95 μg N L^−1^. All data are summarised in Table 1.

### 3.3 PCA of Catchment and Environmental Variables

**FIGURE 4:**
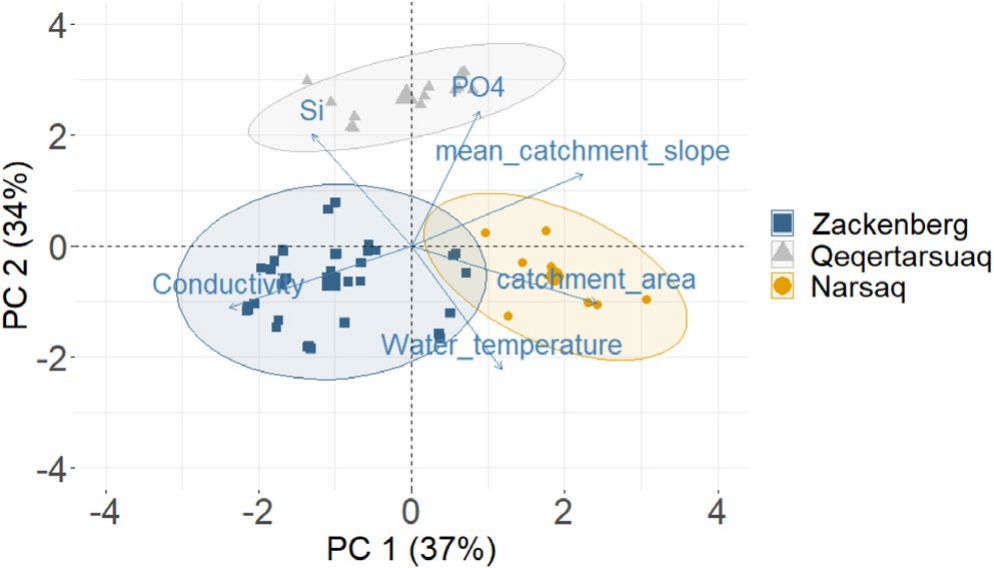
Principal component analysis of environmental and catchment variables. Axis labels indicate percentage of variance explained by the principal components (PC) 1 and 2. Colours and shapes show the observations within Zackenberg (blue square), Qeqertarsuaq (grey triangle), and Narsaq (orange circle).

The first two principal components (PC) axes explained most of the variance in environmental and catchment factors (71%, Figure 4). There was a clear separate clustering of the three regions. Narsaq clustered on the right end of PC 1 and in the middle of PC 2 and was primarily associated with a larger catchment area and steeper catchment slopes (Table S3). Qeqertarsuaq clustered in the higher end of PC 2 and in the middle of PC 1 and was associated with a higher concentration of phosphate and silica. Finally, Zackenberg clustered more towards the left side of the centre and was associated with higher water conductivity. Overall, this emphasises that the three regions represent distinct environmental and catchment settings.

### 3.4 Biofilm Characteristics and Major Autotrophic Groups

**FIGURE 5:**
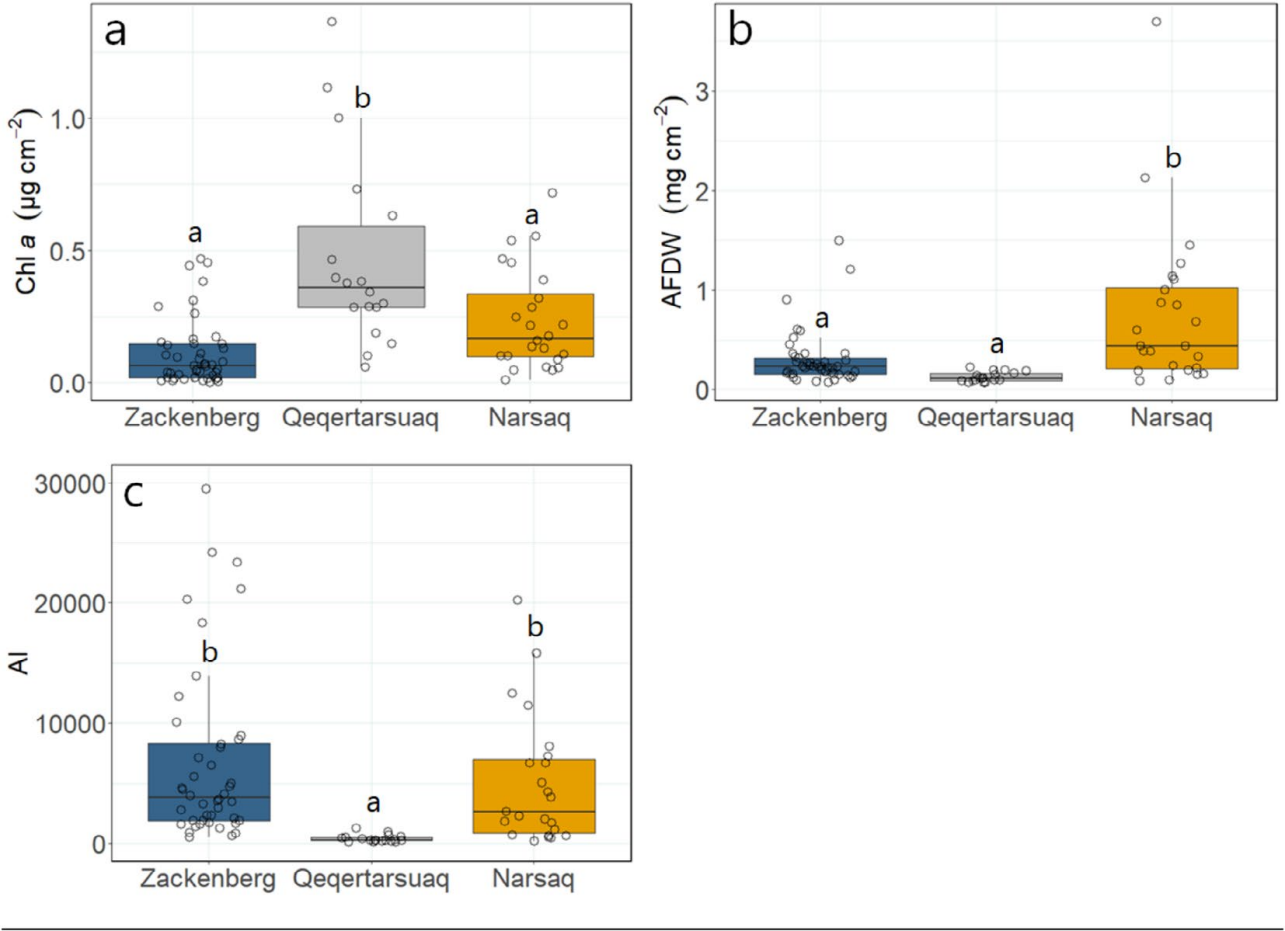
(a) Chl *a* (μg cm^−2^) concentration, (b) amount of AFDW (mg cm^−2^) and (c) AI in streams within the three regions. From left to right, the regions decrease in latitude. One outlier was removed from Narsaq in the AI figure for readability of the figure. Lowercase letters above box plots indicate significant differences (*p* < 0.05) based on Tukey's post hoc analysis.

Epilithic biofilm Chl *a* concentrations were higher in Qeqertarsuaq streams (0.10–1.16 μg cm^−2^, Table S2; Figure 5a) than in Narsaq and Zackenberg (0.04–0.52 and 0.01–0.36 μg cm^−2^, respectively; ANOVA; *F* = 18.38; *p* = 0.0018 and < 0.0001, respectively; Figure 5a; Table S2). The AFDW in Narsaq (0.46–1.57 mg cm^−2^) was greater than in Qeqertarsuaq and Zackenberg (0.08–0.18 and 0.14–1.20 mg cm^−2^, respectively; *F* = 10.72; *p* = 0.0001 and 0.0009, respectively; Figure 5b). The biofilms' AI in Qeqertarsuaq (160–984) was lower than that in Narsaq and Zackenberg (3144–28,744 and 1268–20,588, respectively; *F* = 3.83; *p* = 0.032 and 0.044, respectively; Figure 5c), due to the lowest AFDW and highest Chl *a* values in Qeqertarsuaq.

**FIGURE 6:**
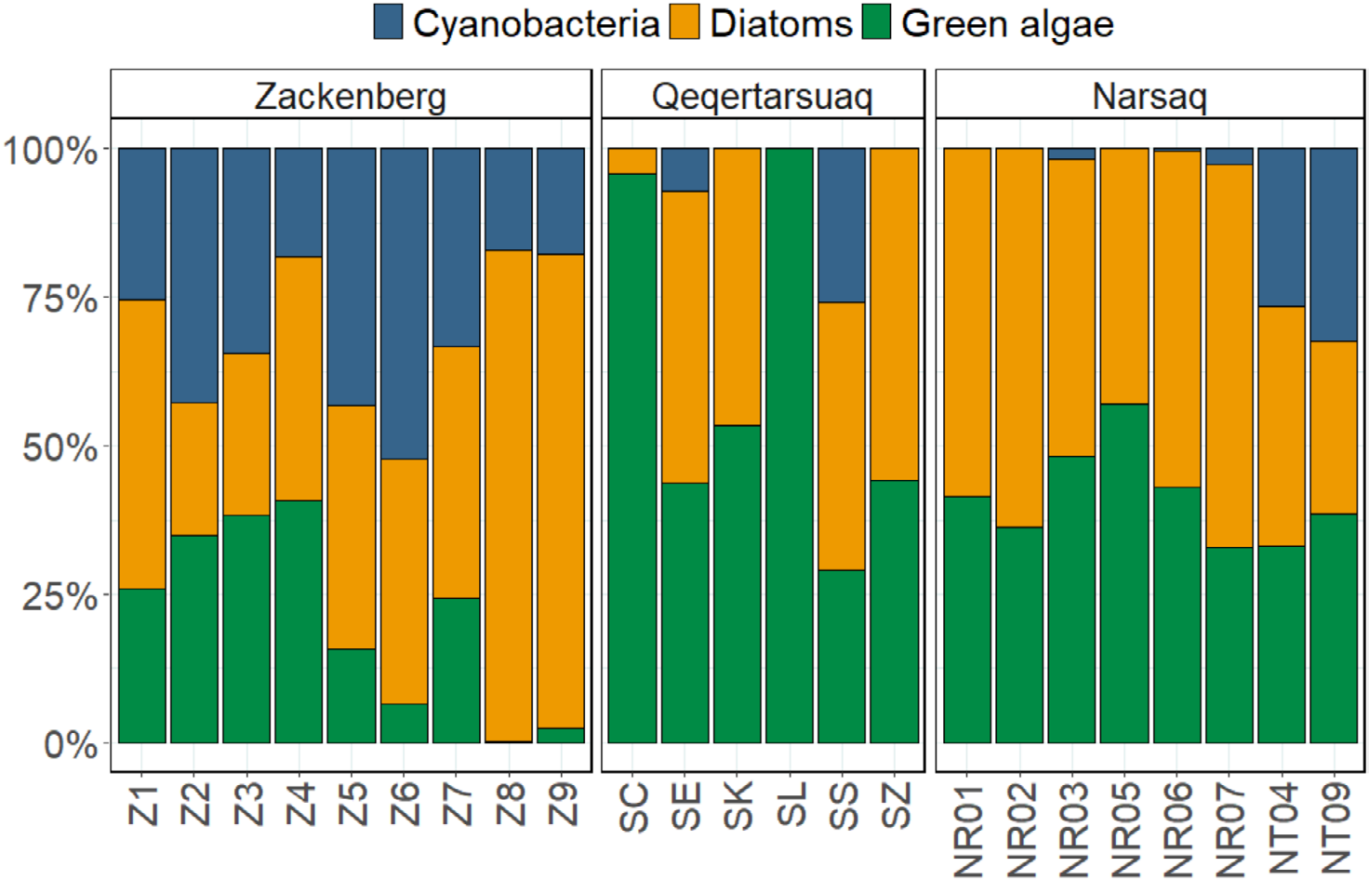
Mean relative abundance of epilithic cyanobacteria, diatoms and green algae at different sites in Narsaq (August 2022), Qeqertarsuaq (July 2023), and Zackenberg (August 2023) based on in situ pigment readings by BenthoTorch (*N* = 9 for each site). Measurements from Qeqertarsuaq and Zackenberg were taken during different sampling campaigns and some at different sites than the other data from these regions (see Section 2). Sites are ordered numerically (Zackenberg) and alphabetically (Qeqertarsuaq and Narsaq).

Cyanobacteria, diatoms and green algae appeared in all regions but with different patterns (Figure 6). In Narsaq, diatoms and green algae made up the majority of autotrophic biomass. However, in the two lake‐fed streams (NT04 and NT09), cyanobacteria contributed with > 25% to total autotrophic biomass. In Qeqertarsuaq, two streams (SC and SL) were dominated by green algae (95%–100%) while in the other streams, green algae and diatoms each formed an important part of the autotrophic biomass. Biofilms in streams SE and SS also contained cyanobacteria. In Zackenberg, cyanobacteria are more predominant than in Narsaq and Qeqertarsuaq, as they are present in all nine streams and make up 15%–55% of total autotrophic biomass. Green algae were less abundant in streams Z5, Z6, Z8 and Z9, contributing < 15% to total biomass.

### 3.5 Spearman Rank Correlations

Spearman correlation coefficients among biofilm, physical–chemical and environmental attributes across the three different regions are shown in Table 2. Values of Chl *a* concentrations were positively correlated with values of phosphate, catchment slope, silica, nitrate, Julian day and current velocity and negatively with iron, latitude, ammonium and water conductivity. Values of AFDW were positively correlated with water temperature, iron, catchment area and velocity and negatively with Julian day, silica and phosphate. The AI was positively correlated with iron, water temperature, ammonium, latitude and catchment area and negatively with silica, phosphate, Julian day and catchment slope. We also did a Spearman correlation for each region separately (Tables S4–S6). This analysis showed that the most important variables correlating with biofilm characteristics varied between regions. Chl *a* concentrations in Zackenberg were positively correlated with velocity, nitrate and Julian day. In Qeqertarsuaq, Chl *a* was positively correlated with velocity, water temperature, Julian day and catchment slope, and in Narsaq positively with conductivity and silica. In Zackenberg, AFDW was positively correlated with nitrate, while in Qeqertarsuaq, it was correlated with velocity, water temperature and catchment slope. AFDW was negatively correlated with conductivity in Qeqertarsuaq. In Narsaq, AFDW was not correlated with any variable. Finally, AI in Zackenberg was positively correlated with NDVI and negatively with velocity, phosphate, Julian day and catchment slope. In Qeqertarsuaq, AI was negatively correlated with velocity, phosphate, nitrate and catchment slope. In Narsaq, AI was not correlated with any variable.

**TABLE 2:** Spearman correlation coefficients for biofilm characteristics and environmental and catchment variables across all sites in Greenland.

	Chl *a*	AFDW	AI	Latitude	Velocity	Water temperature	Conductivity	NH_4_^+^	PO_4_^3−^	NO_3_^−^	Si	Julian day	Catchment NDVI	Catchment area	Catchment slope
**Chl *a***		ns	Not relevant	−0.42	0.26	ns	−0.32	−0.36	0.45	0.29	0.39	0.28	ns	ns	0.39
**AFDW**			Not relevant	ns	0.32	0.45	ns	ns	−0.22	ns	−0.48	−0.51	ns	0.39	ns
**AI**				0.24	ns	0.30	ns	0.24	−0.56	ns	−0.56	−0.55	ns	0.23	−0.36
Latitude					−0.41	ns	0.76	0.74	−0.41	ns	0.82	0.40	ns	−0.51	−0.76
Velocity						0.43	−0.48	−0.22	ns	ns	−0.50	−0.36	−0.29	0.43	0.41
Water temp							ns	ns	−0.25	ns	−0.37	−0.64	ns	0.56	ns
Conductivity								0.65	−0.22	0.27	0.92	0.24	ns	−0.45	−0.79
NH_4_^+^									ns	0.23	0.69	0.23	ns	−0.31	−0.56
PO_4_^3−^										ns	0.45	0.29	0.24	ns	0.36
NO_3_^−^											ns	ns	ns	−0.39	ns
Si												0.52	0.37	−0.64	−0.39
Julian day													ns	−0.75	ns
NDVI														ns	ns
Catchment area															ns
Catchment slope															

*Note:* Positive correlation coefficients are shown in blue and negative correlations are in red. The biofilm response variables are marked in bold. ns = not significant (*p* > 0.05). Coefficients for AI–Chl *a* and AI–AFDW are not relevant due to autocorrelation.

### 3.6 Relationships Between Biofilm Characteristics and Environmental and Catchment Variables

**FIGURE 7:**
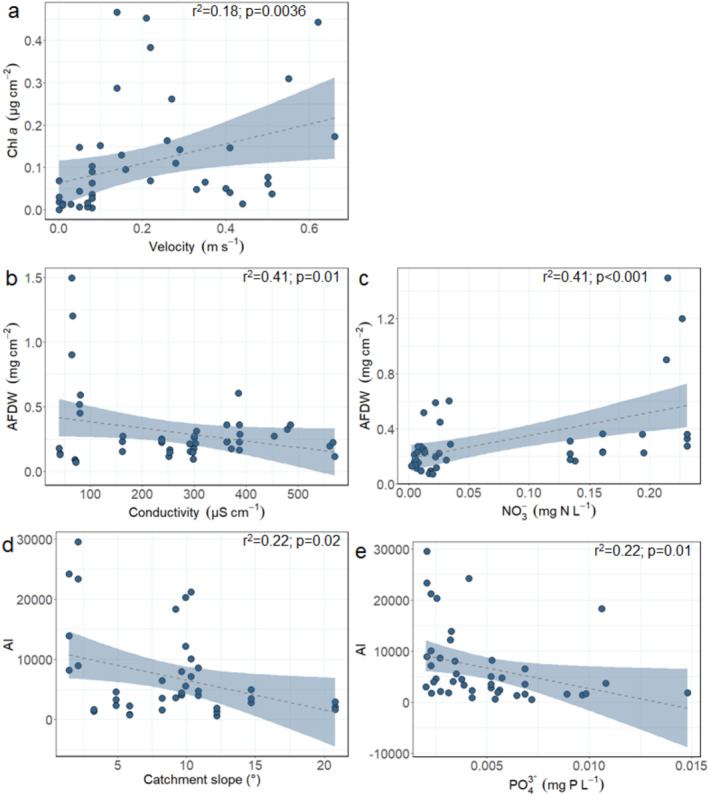
Linear regressions between Zackenberg biofilm: (a) Chl *a* (μg cm^−2^) and stream velocity (m s^−1^; LM; *p* = 0.0036; *F* = 9.58), (b) AFDW (mg cm^−2^) and water conductivity (μS cm^−2^; LM; *p* = 0.01; *F* = 6.56), (c) AFDW (mg cm^−2^) and nitrate concentrations (mg N L^−1^; LM; *p* < 0.001; *F* = 23.55), (d) AI and catchment slope (°; LM; *p* = 0.02; *F* = 6.03) and (e) AI and phosphate concentrations (mg P L^−1^; LM; *p* = 0.01; *F* = 7.40). Dashed lines and blue confidence intervals show significant regressions.

**FIGURE 8:**
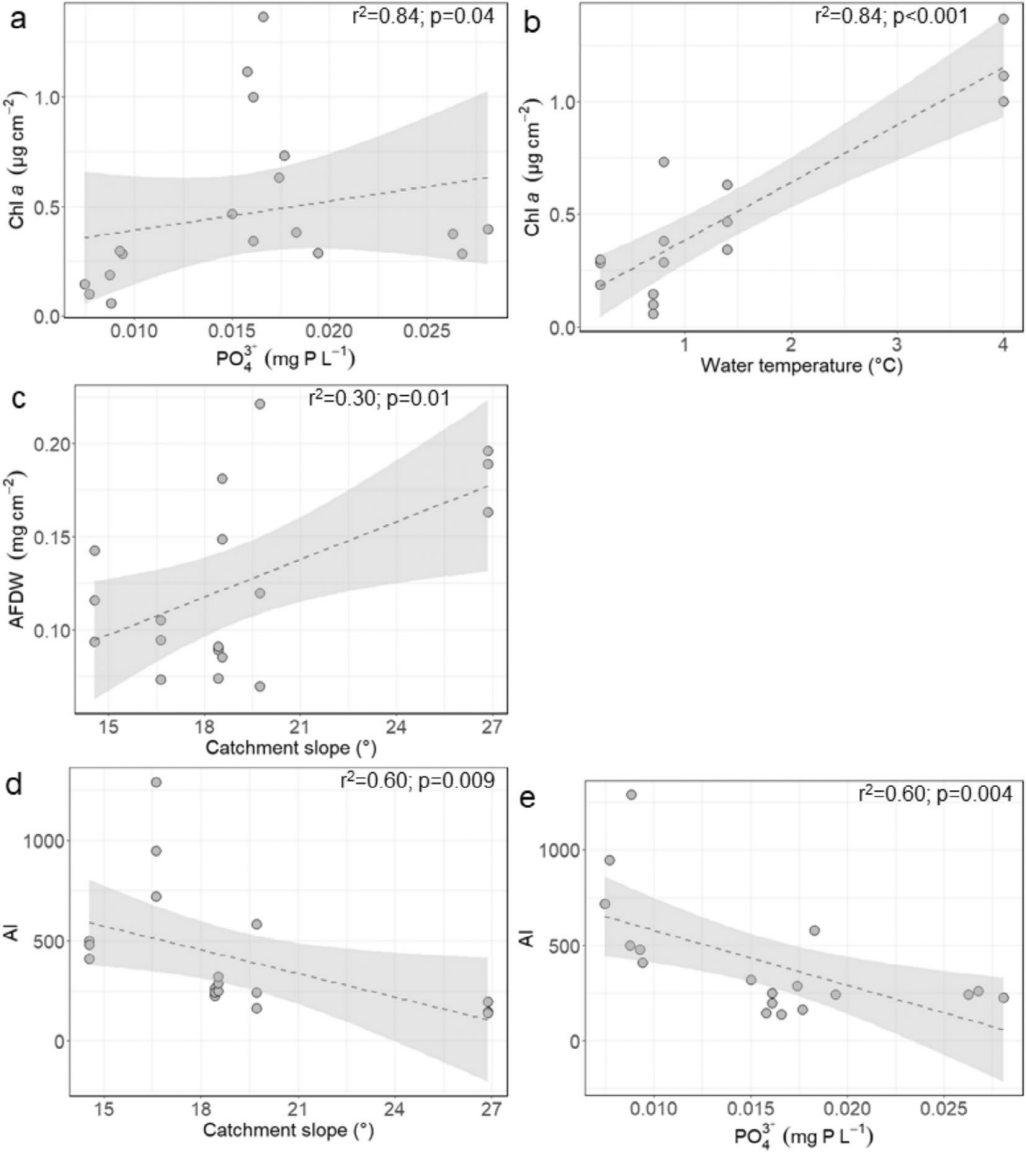
Linear regressions between Qeqertarsuaq biofilm: (a) Chl *a* (μg cm^−2^) and phosphate concentrations (mg P L^−1^; LM; *p* = 0.04; *F* = 5.56), (b) Chl *a* (μg cm^−2^) and water temperature (°C; LM; *p* < 0.001; *F* = 68.40), (c) AFDW (mg cm^−2^) and catchment slope (°; LM; *p* = 0.01; *F* = 7.57), (d) AI and catchment slope (°; LM; *p* = 0.009; *F* = 10.27) and (e) AI and phosphate concentrations (mg P L^−1^; LM; *p* = 0.004; *F* = 13.45). Dashed lines and grey confidence intervals show significant regressions.

**FIGURE 9:**
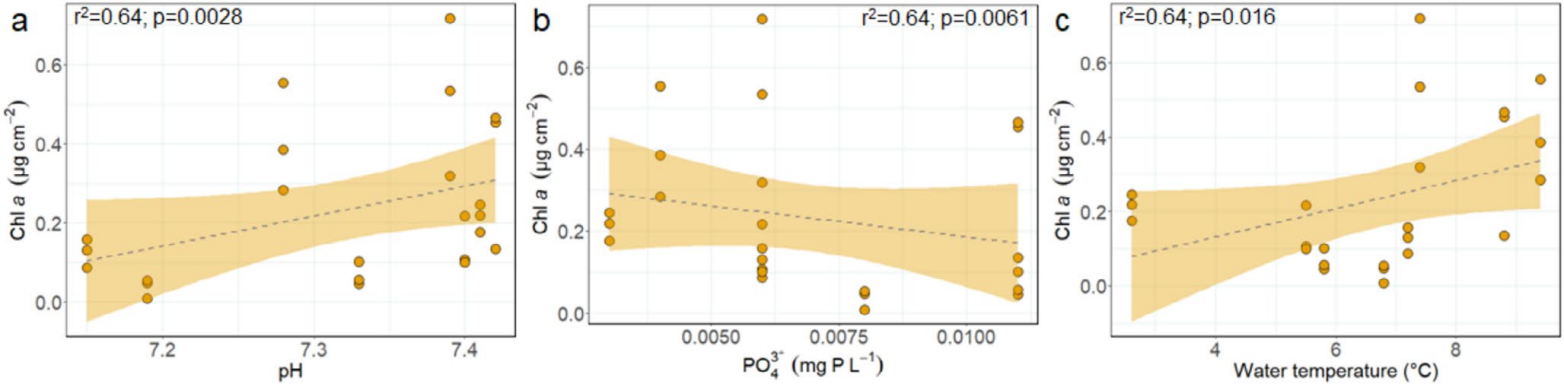
Linear regressions between Narsaq biofilm: (a) Chl *a* (μg cm^−2^) and pH (LM; *p* = 0.0028; *F* = 14.01), (b) Chl *a* (μg cm^−2^) and phosphate concentrations (mg P L^−1^; LM; *p* = 0.0061; *F* = 11.01) and (c) Chl *a* (μg cm^−2^) and water temperature (°C; LM; *p* = 0.016; *F* = 7.80). Dashed lines and orange confidence intervals show significant regressions.

When analysing the data in a multiple linear regression analysis for each region, we found generally corresponding results to the spearman rank correlation. In Zackenberg, Chl *a* concentrations were positively related to stream velocity (Table 3; *F* = 9.58; *p* = 0.0036; Figure 7a), AFDW was negatively related to water conductivity (Figure 7b; *F* = 6.56; *p* = 0.01) and positively to nitrate concentrations (Figure 7c; *F* = 23.55; *p* < 0.001), and AI was negatively related to catchment slope (Figure 7d; *F* = 6.03; *p* = 0.02) and phosphate concentrations (Figure 7e; *F* = 7.40; *p* = 0.01). In Qeqertarsuaq, Chl *a* concentrations were positively related to phosphate concentrations (Figure 8a; *F* = 5.56; *p* = 0.04) and water temperature (Figure 8b; *F* = 68.40; *p* < 0.001), AFDW was positively related to catchment slope (Figure 8c; *F* = 7.57; *p* = 0.01), and AI was positively related to catchment slope (Figure 8d; *F* = 10.27; *p* = 0.009) and negatively to phosphate concentrations (Figure 8e; F = 13.35; *p* = 0.004). Finally, in Narsaq, Chl *a* concentrations were positively related to pH (Figure 9a; *F* = 14.01; *p* = 0.0028) and water temperature (Figure 9c; *F* = 7.80; *p* = 0.016), and negatively to phosphate concentrations (Figure 9b; *F* = 11.01; *p* = 0.0061). There were no significant models for AFDW and AI.

**TABLE 3:** Multiple linear regression models relating biofilm characteristics to physical–chemical, catchment, and environmental variables.

		Estimate	*t*	*F*	*p*	Adj. *R* ^2^	Model sign.	*df*
Zackenberg								
Chl *a*	Catchment area	0.05	0.63	1.56	0.22	0.18	0.007	2, 39
	Velocity	4.15	3.10	9.58	0.0036**			
AFDW	Catchment slope	−0.01	−0.97	0.86	0.36	0.41	< 0.001	3, 38
	Conductivity	−0.00	−3.49	6.56	0.01*			
	NO_3_^−^	4.20	4.85	23.55	< 0.001***			
AI	Catchment slope	−592.6	−2.91	6.03	0.02*	0.22	0.003	2, 38
	PO_4_^3−^	−11e5	−2.72	7.40	0.01*			
Qeqertarsuaq								
Chl *a*	Conductivity	0.01	2.01	0.64	0.44	0.84	< 0.001	4, 10
	PO_4_^3−^	53.18	2.41	5.56	0.04*			
	Velocity	−1.27	−0.75	0.56	0.47			
	Water temperature	0.37	2.01	68.40	< 0.001***			
AFDW	Catchment slope	0.00	1.69	7.57	0.01*	0.30	0.03	2, 15
	Conductivity	−0.00	−1.35	1.81	0.20			
AI	Catchment slope	25.91	0.34	10.27	0.009**	0.60	0.009	4, 10
	Conductivity	−23.41	−2.02	0.57	0.47			
	PO_4_^3−^	−10e4	−3.71	13.35	0.004**			
	Water temperature	−141.26	−0.69	0.48	0.51			
Narsaq								
Chl *a*	Conductivity	0.01	1.27	2.15	0.17	0.64	0.0027	5, 12
	pH	1.18	1.58	14.01	0.0028**			
	PO_4_^3−^	−37.75	−2.81	11.01	0.0061**			
	Velocity	0.18	−0.50	0.25	0.62			
	Water temperature	0.03	0.85	7.80	0.016*			
AFDW	No significant model							
AI	No significant model							

*Note:* Data from all streams are included (*n* = 14 in Zackenberg, *n* = 18 in Qeqertarsuaq, and *n* = 24 in Narsaq).

*p ≤ 0.05; **p ≤ 0.01; ***p ≤ 0.001.

## 4 Discussion

Our goal was to describe biofilm biomass and the relative abundance of major autotrophic groups, as well as evaluate how environmental drivers shape stream epilithic biofilms across three distinct climate regimes representing subarctic, Low Arctic and High Arctic zones in Greenland. In accordance with our initial hypotheses, we found that epilithic stream biofilm in the three study areas primarily related to water temperature and nutrient concentrations (Table 2). Other important variables were water conductivity and catchment characteristics.

As temperatures are projected to increase during the next decades (Rantanen et al. 2022), which will likely also promote increases in nutrient levels across the Arctic landscape (Elberling et al. 2008; Vonk et al. 2015), we expect that biofilm biomass will have the potential to increase. However, to which extent this will affect the ecosystem structure and function will depend on several factors, such as light availability, nutrient availability and grazing pressure. In the following, we will first characterise the stream biofilm in the three regions and then discuss how these results support our hypotheses that (1) climatic conditions (e.g., temperature) drive biofilm autotrophic and total biomass; (2) catchment size, slope and vegetation cover positively affect biofilm biomass and (3) nutrient availability in the water column affects biofilm autotrophic and total biomass.

### 4.1 Epilithic Biofilm Across Climate Regions in Greenland

The range of Chl *a* concentrations in epilithic stream biofilm in this study based on 28 Greenland stream sites was 0.00–1.36 μg cm^−2^. This is similar to other regions in the Arctic, where Myrstener et al. (2018) reported values of 0.1–1.4 μg cm^−2^ for tundra streams in Arctic Sweden, and Kendrick and Huryn (2015) reported values of 0.25–0.69 μg cm^−2^ from streams in Alaska. The streams in Qeqertarsuaq exhibited significantly higher Chl *a* concentrations than those in the Narsaq and Zackenberg regions. One primary reason for this difference could be that the Qeqertarsuaq sites were sampled later during the growing season than the two other regions, and therefore, provided more time for biomass accumulation over the season. This is supported by Hauptmann and Myrstener (2023) that showed that biofilm Chl *a* concentrations increased throughout the season in streams in Arctic Sweden. However, Skovsholt et al. (2020) found that in Zackenberg streams, autotrophic biomass was higher in early summer and total biomass in late summer, while primary production was higher in late summer compared to early summer. They linked this to the lower discharge and thus lower physical disturbance, allowing for biomass accumulation (Skovsholt et al. 2020). The occurrence of geothermal springs in the Qeqertarsuaq area allows for year‐round flowing water in some of the study streams (SE, SK and SS), extending the length of the growing season (Friberg et al. 2001; Hjartarson and Armannsson 2010). Thus, the higher Chl *a* concentrations in Qeqertarsuaq are likely the result of a combination of warmer streams, a longer growing season, and sampling later in the season.

Our results show that total biofilm biomass (AFDW) was positively correlated with water temperature across the three climate regions (Table 2) and that Chl *a* concentrations were positively related to water temperature in Qeqertarsuaq and Narsaq (Table 3). Therefore, hypothesis 1—that temperature drives both autotrophic and total biomass—was verified. A warming experiment in the subarctic also showed that higher water temperatures resulted in increased autotrophic biomass, primarily due to an increase in green algal biomass, which was related to higher uptake rates of dissolved inorganic nitrogen (Hood et al. 2018). At the cellular level, respiration generally has a higher temperature sensitivity than photosynthesis (Allen et al. 2005; Gillooly et al. 2001). This higher temperature sensitivity of respiration relative to photosynthesis could promote heterotrophy with warming (Allen et al. 2005; Yvon‐Durocher et al. 2010). Accordingly, in our study, we observed that AI positively correlated with temperature across regions, indicating a higher relative importance of the heterotrophic community over the autotrophic one. However, this relation did not hold when examining the regions separately, suggesting that other environmental variables, such as resource supply, could play an overlapping role in controlling the biofilm metabolic balance (Cross et al. 2022; Welter et al. 2015). Finally, the light climate varies significantly between these regions, with Zackenberg experiencing long polar days (24‐h daylight) during summer, while Narsaq has nights where the sun sets. This difference is likely to affect growth and, consequently, biofilm biomass (Bernhardt et al. 2022; Von Schiller et al. 2007).

Stream epilithic biofilms across the three studied regions consisted of a high relative proportion of heterotrophic organisms and non‐living organic material, as shown by the AI > 200 (Steinman et al. 2017). Qeqertarsuaq had lower AI values than Narsaq and Zackenberg, in accordance with the higher autotrophic biomass and relatively low total organic biomass. Heterotrophic organisms utilise organic material and inorganic nutrients for growth, while autotrophs are dependent on inorganic nutrients and light for production (Quesada et al. 2008) and therefore the results suggest that streams in the Narsaq and Zackenberg areas are more nutrient limited than the Qeqertarsuaq streams. This corresponds with higher phosphate concentrations in Qeqertarsuaq (Table 1; Figure 4), which could be related to the area's distinctive lithology (e.g., basaltic rock; Larsen and Larsen 2022; Porder and Ramachandran 2013). Thus, in addition to the effect of warmer streams, a longer growing season and the timing of sampling, as discussed above, the higher Chl *a* concentrations in Qeqertarsuaq are likely also the result of higher phosphate concentrations.

It is important to emphasise that the three regions included in this study differed markedly from each other in terms of their physical–chemical and environmental conditions (Table 1). The sites in Qeqertarsuaq especially deviated from Narsaq and Zackenberg, both in terms of the conditions and biofilm characteristics. The PCA showed a general separation of the three regions based on environmental and catchment variables (Figure 4). This might partially be explained by the fact that the time of sampling differed and that the timing in the summer hydrological patterns can vary per season, but studies with continuous biofilm sampling across the summer season are needed to test this. Assessing temporal dynamics in Arctic streams is challenging but essential to fully understand microbial biofilm dynamics. Temporal metrics are difficult to standardise: should timing be assessed by specific dates, days from the onset of thaw, or degree days (cumulative temperature), each of which has distinct implications for interpreting microbial growth and environmental dynamics (Rasmussen et al. 2022)? Degree days may offer a standardised approach by capturing cumulative temperature exposure, yet date‐based timing can provide a valuable link to broader seasonal and hydrological cycles. In the Arctic, sampling frequently is often logistically challenging due to remote site access and limited seasonal windows, yet this information is necessary to capture the full seasonal progression of biofilm development and microbial community responses to environmental shifts.

### 4.2 Biofilm Topographic and Environmental Controls

The significance of nutrient limitation is paramount in biofilm accrual in Arctic streams (Docherty, Riis, Milner, et al. 2018; Myrstener et al. 2018). We found that Chl *a* concentrations in Qeqertarsuaq were positively related to phosphate concentrations and that AI in Zackenberg and Qeqertarsuaq was negatively related to phosphate concentrations. This could indicate that epilithic autotrophic production is phosphate‐limited in these two regions. However, during an in situ nutrient‐uptake experiment, Docherty, Riis, Milner, et al. (2018) found that nitrogen was the primary limiting nutrient in Zackenberg streams, while phosphate limitation was more widespread. In Narsaq, there was a negative relation between Chl *a* and phosphate concentrations (Table 3). All stream sites in Narsaq were nitrogen‐limited (Table 1), potentially explaining this relation between Chl *a* and phosphate. Additionally, we found a positive relation between nitrate concentrations and AFDW in Zackenberg. Thus, hypothesis 3—that the availability of inorganic nutrients in the water column affects biofilm biomass—was verified. Nutrient availability was generally low, with several ammonium and phosphate levels being under the detection limit (Table 1). However, the microalgae in the multi‐organism assemblage may also utilise nutrients recycled within the biofilm mat (Allan et al. 2021) thus making the coupling between water column nutrients and biomass less strong. Based on our three very different regions in Greenland, spanning from subarctic to High Arctic, these positive relations with phosphate and nitrate, in our Low Arctic and High Arctic site, indicate that these stream biofilms are overall nutrient limited, confirming results from other studies in Greenland and other Arctic regions (Docherty, Riis, Hannah, et al. 2018; Hauptmann and Myrstener 2023; Kendrick and Huryn 2015).

The large share of cyanobacteria in biofilm communities, such as in Zackenberg, is often seen as a sign of nitrogen limitation, as some species are capable of nitrogen fixation (Diehl et al. 2018). However, both ammonium and nitrate concentrations (also relative to phosphate) in Zackenberg were generally larger than in the two other regions (Table 1). During their nutrient uptake experiments, Docherty, Riis, Milner, et al. (2018) showed that, despite the fact that streams in Zackenberg displayed a widespread phosphate limitation, nitrogen was found to be the primary limiting nutrient for biofilm. Another study from Zackenberg (Pastor et al. 2020) also showed a nitrogen limitation within biofilm during an experiment with nutrient‐diffusing substrata. This would explain the presence of cyanobacteria in the Zackenberg streams. In addition, Von Friesen et al. (2023) confirmed the presence of pelagic cyanobacteria in river outlets in Zackenberg but emphasised that more research to fully understand the role of cyanobacteria in these areas is needed. Furthermore, this suggests that nutrient concentrations in stream water might not always be indicative of the nutrient availability for biofilms.

Our second hypothesis—that topographic and watershed characteristics affect biofilm biomass—was only partially accepted, because catchment NDVI was not related to any of the biofilm characteristics. However, catchment slope was negatively related to AI in Zackenberg and positively to AFDW and AI in Qeqertarsuaq (Table 3). This is supported by previous studies showing that nitrate concentrations in streams were positively associated with catchment slope (Connolly et al. 2018; Harms et al. 2016) and thus may be an indirect driver of biofilm biomass. Higher catchment slope may be linked to higher erosion rates and thus higher runoff of geological or soil‐derived nutrients from catchment to stream as well as short water travel time. Since biofilms are nutrient limited, as discussed above, this suggests that higher nutrient levels due to higher slope promote biofilm accumulation. Furthermore, the observed significant negative correlation between AI and catchment slope (Table 3) would confirm that the proportion of autotrophs to total biofilm biomass increases with increasing slope of the catchment. Although NDVI has shown to be negatively correlated to high nitrate concentration in headwater streams in Zackenberg (Riis et al. 2023), NDVI did not correlate with biofilm biomass both when analysing across all three regions and when analysing within regions. Identifying master variables that can be obtained from satellites or maps (e.g., catchment slope) to extrapolate results across remote Arctic areas, where field data on water chemistry are unavailable, can improve our ability to assess and predict stream biofilm dynamics in relation to watershed nutrient export (Connolly et al. 2018).

In this study, the impact of grazers on biofilms was not included. However, grazers living in biofilms, such as ciliates, nematodes, and chironomids, can significantly change biofilm community composition, physical structure, and the cycling of carbon (Battin et al. 2016; Hakenkamp and Morin 2000; Lawrence et al. 2002). Thus, grazing is an important biological driver of biofilm biomass, but the quantitative effect was beyond the scope of this study.

### 4.3 Potential Effects of Global Change

As annual temperatures in the Arctic are projected to increase further in the coming decades (Rantanen et al. 2022), our results suggest that epilithic biofilm biomass will increase, based on the correlation with temperature per se. A similar relationship between temperature and biofilm accumulation in global glacier streams was also found by Kohler et al. (2024). Furthermore, aquatic nutrient concentrations are expected to increase in some regions as a result of permafrost thaw (Vonk et al. 2015). This will also lead to a greater autotrophic and total biofilm biomass as biofilms are currently nutrient limited (Docherty, Riis, Hannah, et al. 2018; Hauptmann and Myrstener 2023). Contrastingly, the expected increase of precipitation in the Arctic (McCrystall et al. 2021), mostly as rainfall (Bintanja and Andry 2017), could have a negative impact on biofilm accumulation, as biofilm biomass is lower at high flow conditions (Battin et al. 2003; Rinke et al. 2001). In particular, heavy rainfall events in summer (Beel et al. 2021) and more heterogeneous discharge (Feng et al. 2021) in the Arctic could lead to biofilm being washed away. In addition, increased turbidity due to permafrost thaw and increased inflow of dissolved organic carbon (Kokelj et al. 2009) may reduce light availability and therefore negatively impact biofilm biomass. Future research should focus on including more streams and regions within the circumpolar Arctic and on the role that food web interactions play in the functioning and community composition of epilithic biofilm.

## 5 Conclusion

Our study characterised stream biofilm in the Arctic across a large geographical scale (60°–74° N) in Greenland, including subarctic, Low and High Arctic, and identified the main drivers. Biofilms in Qeqertarsuaq differed from the ones in Narsaq and Zackenberg by their high Chl *a* concentration and low AFDW, while the relative abundance of major autotrophic groups in Zackenberg differed from Narsaq and Qeqertarsuaq due to the relatively high abundance of cyanobacteria. Across sampling streams, we showed that epilithic biofilms related to nutrient availability and catchment topography. Future warming may result in increased organic carbon and nutrient availability in the streams, which then again will promote biofilm biomass accumulation, causing alterations to trophic food webs and biogeochemical cycling. However, the extent of these effects will also depend on light availability, which might also change as a result of increased runoff. Further research is warranted to increase the understanding of how stream biofilm in Greenland and the further circumpolar Arctic is shaped through its surroundings.

## Author Contributions

**Sanne M. Moedt:** conceptualization, methodology, formal analysis, investigation, visualization, writing – original draft, writing – review and editing. **Kirsten S. Christoffersen:** conceptualization, methodology, investigation, formal analysis, supervision, funding acquisition, project administration, resources, writing – review and editing. **Andreas Westergaard‐Nielsen:** formal analysis, visualization, writing – review and editing, methodology. **Kenneth T. Martinsen:** formal analysis, writing – review and editing, methodology. **Ada Pastor:** conceptualization, methodology, writing – review and editing. **Niels Jákup Korsgaard:** formal analysis, methodology, writing – review and editing. **Tenna Riis:** conceptualization, methodology, formal analysis, investigation, supervision, funding acquisition, project administration, resources, writing – review and editing.

## Conflicts of Interest

The authors declare no conflicts of interest.

## Supporting information


Data S1.

## Data Availability

Climate data were obtained from the Greenland Ecosystem Monitoring and Danish Meteorological Institute databases. Environmental and biofilm data used in the paper are available in Moedt et al. (2024).
