# Supplementary material for: Drivers of Epilithic Biofilms in Greenland Streams: The Role of Nutrients, Temperature and Catchment Slope Across a Climate Gradient
Source: Environ Microbiol Rep. 2025 Mar 12;17(2):e70074. doi: 10.1111/1758-2229.70074 (PMC11903329; doi:10.1111/1758-2229.70074)
Supplement: Supplementary file 1 — Data S1. [file EMI4-17-e70074-s001.docx]

**Supporting information**

**Drivers of Epilithic Biofilms in Greenland Streams: The Role of Nutrients, Temperature, and Catchment Slope across a Climate Gradient**

**Running title:** *Drivers of Epilithic Biofilms in Greenland Streams*

**Sanne M. Moedt^1^, Kirsten S. Christoffersen^1,2^, Andreas Westergaard-Nielsen^3^, Kenneth T. Martinsen^1^, Ada Pastor^4^, Niels Jákup Korsgaard^5^, and Tenna Riis^6^**

^1^Department of Biology, University of Copenhagen, Copenhagen, Denmark

^2^Arctic Biology, University Centre in Svalbard, Svalbard, Norway

^3^Department of Geosciences and Natural Resource Management, University of Copenhagen, Copenhagen, Denmark

^4^Institute of Aquatic Ecology, University of Girona, Girona, Spain

^5^Geological Survey of Denmark and Greenland, Copenhagen, Denmark

^6^Department of Biology, Arctic Research Center, Aarhus University, Aarhus, Denmark

**Table S1.** Coordinates and sampling date of sampling sites in Zackenberg, Qeqertarsuaq, and Narsaq.

| Region | Stream | Sampling date (dd-mm-yyyy) | Latitude | Longitude |
| --- | --- | --- | --- | --- |
| Zackenberg | AE | 30-08-2017 | 74°30'11.2"N | 20°32'36.1"W |
|  | GA | 27-08-2017 | 74°29'14.6"N | 20°29'02.9"W |
|  | GB | 27-08-2017 | 74°28'50.1"N | 20°29'47.7"W |
|  | GC | 25-08-2017 | 74°28'23.6"N | 20°30'30.0"W |
|  | KO | 24-08-2017 | 74°29'52.5"N | 20°28'57.8"W |
|  | KA | 24-08-2017 | 74°29'40.9"N | 20°29'46.6"W |
|  | KB | 21-08-2017 | 74°29'01.8"N | 20°32'05.6"W |
|  | KC | 20-08-2017 | 74°28'15.7"N | 20°31'07.8"W |
|  | LT | 30-08-2017 | 74°32'21.1"N | 20°39'12.3"W |
|  | P | 30-08-2017 | 74°31'38.2"N | 20°34'30.7"W |
|  | S1 | 31-08-2017 | 74°29'26.3"N | 20°35'16.9"W |
|  | S2 | 31-08-2017 | 74°28'02.2"N | 20°39'12.3"W |
|  | S3 | 31-08-2017 | 74°28'17.9"N | 20°36'31.1"W |
|  | U | 30-08-2017 | 74°30'16.8"N | 20°33'43.6"W |
| Qeqertarsuaq | A | 22-09-2018 | 69°16'27.9"N | 53°29'25.9"W |
|  | B | 22-09-2018 | 69°15'40.5"N | 53°29'50.2"W |
|  | E | 25-09-2018 | 69°15'38.9"N | 53°34'09.1"W |
|  | K | 18-09-2018 | 69°15'51.1"N | 53°26'43.2"W |
|  | L | 18-09-2018 | 69°15'60.0"N | 53°30'34.7"W |
|  | S | 22-09-2018 | 69°15'25.2"N | 53°31'18.2"W |
| Narsaq | NR03 | 04-08-2022 | 60°57'20.3"N | 46°00'30.6"W |
|  | NR05 | 04-08-2022 | 60°57'08.4"N | 46°01'02.2"W |
|  | NR06 | 04-08-2022 | 60°57'00.4"N | 46°01'28.2"W |
|  | NT04 | 04-08-2022 | 60°56'54.9"N | 46°01'04.5"W |
|  | NR07 | 05-08-2022 | 60°56'22.4"N | 46°02'28.1"W |
|  | NR01 | 06-08-2022 | 60°58'50.5"N | 45°57'00.5"W |
|  | NR02 | 06-08-2022 | 60°57'58.7"N | 45°59'07.7"W |
|  | NT09 | 06-08-2022 | 60°57'13.4"N | 46°00'58.2"W |

**Digital elevation model (DEM), Qeqertarsuaq area.**

A 2009 DEM and orthophoto was made from overlapping aerial photos from an aerial survey campaign conducted that year by Scankort A/S (data set later purchased by Fugro). The aerial survey campaign of Disko Island and the Nuussuaq Peninsula was conducted using a commercial-grade Intergraph Z/I DMC digital mapping camera. Eleven digital photos from two strips covers the area surrounding the town of Qeqertarsuaq (strip 15: 1650-1654 and strip 14: 1831-1836) which were recorded on July 18, 2009.

We established Ground Control Points (GCP) by identifying 20 evenly distributed points which could be found in the eleven aerial photos as well as in an orthophoto (and DEM) made from SPOT 6/7 satellite images (SDFI, 2018). Horizontal coordinates in UTM22/WGS84 and elevations above Mean Sea Level were transferred to the GCPs from the satellite data.

The photogrammetric software Bingo 6.2a was used for a weight normalized bundle adjustment with standard deviations x = 1.5 m, y = 0.7 m, and z = 1.9 m (i.e. 1.7 m horizontally and 1.9 m vertically). The exterior camera positions and orientations calculated in Bingo was imported into Socet Set 5.2 where the NGATE module extracted the 2009 DEM in 2 m resolution, and orthophotos were made in both RGB and CIR with a pixel resolution of 0.5 m.

The Spot 6/7 satellite orthophotos and DEM used as reference data to derive coordinates and heights for the GCPs in 2019 were available at a data repository in 2018 (SDFI, 2018). It is no longer available and has been superseded by SDFI (2024a; 2024b). Here it is stated by the producer that the satellite orthoimage has an absolute geometrical accuracy better than 16 m (10 pixel) (SDFI 2024a). The DEM (SDFI, 2024b) is a composite of ArcticDEM, GIMP, and TREx. The latter two data sets have been used for void-filling for surface continuity (SDFI 2024b). As ArcticDEM data is available for Disko Ø it is safe to assume that elevations extracted to the GCPs are derived from the ArcticDEM.

In the summer of 2018 we validated the DEM using 44 independent DGPS-corrected GCPs covering a combination of high and low-lying areas (measured with Trimble R8 in a rover-base setup). The validation points showed a systematic linear shift of 8.6 m towards south, which was corrected. The remaining average uncertainty in x, y was 0.83 m, and 1.19 m for the elevation, z.

SDFI (Agency for Data Supply and Infrastructure): Grønlandske pilotdata – Disko Bugt [DEM] [ORTOFOTO], <https://dataforsyningen.dk/data/4516>, last access: 28 June 2018.

SDFI (Agency for Data Supply and Infrastructure): Satellitfoto Grønland [ORTOFOTO], <https://dataforsyningen.dk/data/4783>, last access: 26 July 2024a.

SDFI (Agency for Data Supply and Infrastructure): Grønland Højdemodel Overflade [DEM] [ORTOFOTO], <https://dataforsyningen.dk/data/4780>, last access: 26 July 2024b.

**Table S2.** Biofilm characteristics at the stream sites: chlorophyll *a* concentration (Chl *a*), organic matter content as ash-free dry weight (AFDW), autotrophic index (AI).

| Region | Stream | Chl *a*  (µg cm^−2^) | AFDW  (mg cm^−2^) | AI |
| --- | --- | --- | --- | --- |
| Zackenberg | AE | 0.30 ± 0.15 | 0.32 ± 0.04 | 1337 ± 845 |
|  | GA | 0.02 ± 0.01 | 0.25 ± 0.10 | 12680 ± 7381 |
|  | GB | 0.03 ± 0.04 | 0.24 ± 0.01 | 10973 ± 10365 |
|  | GC | 0.01 ± 0.00 | 0.14 ± 0.03 | 15441 ± 8085 |
|  | KO | 0.07 ± 0.02 | 0.23 ± 0.07 | 3750 ± 1129 |
|  | KA | 0.09 ± 0.06 | 0.27 ± 0.08 | 3871 ± 2469 |
|  | KB | 0.02 ± 0.02 | 0.21 ± 0.10 | 12788 ± 7406 |
|  | KC | 0.01 ± 0.01 | 0.17 ± 0.04 | 20588 ± 10545 |
|  | LT | 0.04 ± 0.02 | 0.18 ± 0.06 | 5517 ± 2148 |
|  | P | 0.14 ± 0.03 | 0.22 ± 0.06 | 1547 ± 140 |
|  | S1 | 0.36 ± 0.10 | 1.20 ± 0.30 | 3422 ± 1129 |
|  | S2 | 0.07 ± 0.01 | 0.15 ± 0.03 | 2251 ± 635 |
|  | S3 | 0.10 ± 0.03 | 0.52 ± 0.07 | 5786 ± 2481 |
|  | U | 0.30 ± 0.16 | 0.36 ± 0.22 | 1268 ± 649 |
| Qeqertarsuaq | A | 0.25 ± 0.06 | 0.12 ± 0.02 | 462 ± 46 |
|  | B | 0.10 ± 0.04 | 0.09 ± 0.02 | 984 ± 287 |
|  | E | 1.16 ± 0.19 | 0.18 ± 0.02 | 160 ± 31 |
|  | K | 0.35 ± 0.06 | 0.08 ± 0.01 | 243 ± 18 |
|  | L | 0.47 ± 0.23 | 0.14 ± 0.08 | 329 ± 221 |
|  | S | 0.48 ± 0.15 | 0.14 ± 0.05 | 286 ± 35 |
| Narsaq | NR01 | 0.21 ± 0.04 | 0.48 ± 0.54 | 2264 ± 2433 |
|  | NR02 | 0.14 ± 0.07 | 0.67 ± 0.68 | 4087 ± 2494 |
|  | NR03 | 0.07 ± 0.03 | 0.63 ± 0.44 | 10574 ± 8482 |
|  | NR05 | 0.04 ± 0.02 | 0.46 ± 0.32 | 28744 ± 37589 |
|  | NR06 | 0.12 ± 0.04 | 0.83 ± 0.54 | 7115 ± 4908 |
|  | NR07 | 0.52 ± 0.20 | 0.56 ± 0.25 | 1328 ± 1152 |
|  | NT04 | 0.41 ± 0.14 | 1.57 ± 1.88 | 3144 ± 3176 |
|  | NT09 | 0.35 ± 0.19 | 0.81 ± 1.14 | 5487 ± 8947 |

**Table S3.** Loadings of stream environmental variables for PC1 and PC2.

|  | PC 1 | PC 2 |
| --- | --- | --- |
| Catchment area | 0.54 | -0.24 |
| Catchment slope | 0.50 | 0.30 |
| Conductivity | -0.53 | -0.26 |
| PO_4_^3-^ | 0.20 | 0.56 |
| Si | -0.29 | 0.46 |
| Water temperature | 0.26 | -0.51 |

**Table S4.** Spearman correlation coefficients for biofilm characteristics and environmental and catchment variables in Zackenberg. Positive correlation coefficients are shown in blue and negative correlations in red. The biofilm response variables are marked in bold. ns = not significant (P > 0.05). Coefficients for AI – Chl a and AI – AFDW are not relevant due to autocorrelation.

|  | **Chl a** | **AFDW** | **AI** | Velocity | Water temperature | Conductivity | NH_4_^+^ | PO_4_^3-^ | NO_3_^-^ | Julian day | Catchment  NDVI | Catchment area | Catchment slope |
| --- | --- | --- | --- | --- | --- | --- | --- | --- | --- | --- | --- | --- | --- |
| **Chl a** |  | 0.64 | Not relevant | 0.55 | ns | ns | ns | ns | 0.52 | 0.61 | ns | ns | ns |
| **AFDW** |  |  | Not relevant | ns | ns | ns | ns | ns | 0.62 | ns | ns | ns | ns |
| **AI** |  |  |  | -0.61 | ns | ns | ns | -0.48 | ns | -0.51 | 0.58 | ns | -0.23 |
| Velocity |  |  |  |  | ns | ns | ns | ns | ns | 0.67 | -0.84 | ns | ns |
| Water temp |  |  |  |  |  | ns | 0.09 | ns | ns | -0.24 | ns | ns | ns |
| Conductivity |  |  |  |  |  |  | ns | ns | ns | -0.35 | ns | ns | ns |
| NH_4_^+^ |  |  |  |  |  |  |  | ns | ns | ns | ns | ns | ns |
| PO_4_^3-^ |  |  |  |  |  |  |  |  | ns | ns | ns | -0.02 | ns |
| NO_3_^-^ |  |  |  |  |  |  |  |  |  | ns | ns | -0.45 | ns |
| Si |  |  |  |  |  |  |  |  |  | 0.61 | -0.43 | ns | ns |
| Julian day |  |  |  |  |  |  |  |  |  |  | ns | ns | -0.14 |
| Catchment NDVI |  |  |  |  |  |  |  |  |  |  |  | ns | ns |
| Catchment area |  |  |  |  |  |  |  |  |  |  |  |  | ns |
| Catchment slope |  |  |  |  |  |  |  |  |  |  |  |  |  |

**Table S5.** Spearman correlation coefficients for biofilm characteristics and environmental and catchment variables in Qeqertarsuaq. Positive correlation coefficients are shown in blue and negative correlations in red. The biofilm response variables are marked in bold. ns = not significant (P > 0.05). Coefficients for AI – Chl a and AI – AFDW are not relevant due to autocorrelation.

|  | **Chl a** | **AFDW** | **AI** | Velocity | Water temperature | Conductivity | NH_4_^+^ | PO_4_^3-^ | NO_3_^-^ | Si | Julian day | Catchment NDVI | Catchment area | Catchment slope |
| --- | --- | --- | --- | --- | --- | --- | --- | --- | --- | --- | --- | --- | --- | --- |
| **Chl a** |  | 0.65 | Not relevant | 0.59 | 0.86 | ns | ns | ns | ns | ns | 0.51 | ns | ns | 0.86 |
| **AFDW** |  |  | Not relevant | 0.32 | 0.43 | -0.30 | ns | ns | ns | ns | ns | ns | ns | 0.49 |
| **AI** |  |  |  | -0.59 | ns | ns | ns | -0.53 | -0.52 | ns | ns | ns | ns | -0.74 |
| Velocity |  |  |  |  | 0.82 | ns | ns | ns | ns | ns | 0.87 | ns | -0.94 | 0.70 |
| Water temp |  |  |  |  |  | ns | ns | ns | ns | ns | 0.56 | ns | ns | 0.96 |
| Conductivity |  |  |  |  |  |  | ns | ns | ns | 0.48 | ns | 0.32 | ns | -0.81 |
| NH_4_^+^ |  |  |  |  |  |  |  | ns | ns | ns | ns | ns | ns | ns |
| PO_4_^3-^ |  |  |  |  |  |  |  |  | 0.81 | ns | -0.21 | ns | ns | ns |
| NO_3_^-^ |  |  |  |  |  |  |  |  |  | ns | -0.20 | ns | ns | ns |
| Si |  |  |  |  |  |  |  |  |  |  | ns | 0.54 | -0.60 | ns |
| Julian day |  |  |  |  |  |  |  |  |  |  |  | ns | -0.94 | ns |
| Catchment NDVI |  |  |  |  |  |  |  |  |  |  |  |  | -0.81 | ns |
| Catchment area |  |  |  |  |  |  |  |  |  |  |  |  |  | ns |
| Catchment slope |  |  |  |  |  |  |  |  |  |  |  |  |  |  |

**Table S6.** Spearman correlation coefficients for biofilm characteristics and environmental and catchment variables in Narsaq. Positive correlation coefficients are shown in blue and negative correlations in red. The biofilm response variables are marked in bold. ns = not significant (P > 0.05). Coefficients for AI – Chl a and AI – AFDW are not relevant due to autocorrelation.

|  | **Chl a** | **AFDW** | **AI** | Velocity | Water temperature | Conductivity | NH_4_^+^ | PO_4_^3-^ | NO_3_^-^ | Si | Julian day | Catchment  NDVI | Catchment area | Catchment slope |
| --- | --- | --- | --- | --- | --- | --- | --- | --- | --- | --- | --- | --- | --- | --- |
| **Chl a** |  | ns | Not relevant | ns | ns | 0.36 | ns | ns | ns | 0.27 | ns | ns | ns | ns |
| **AFDW** |  |  | Not relevant | ns | ns | ns | ns | ns | ns | ns | ns | ns | ns | ns |
| **AI** |  |  |  | ns | ns | ns | ns | ns | ns | ns | ns | ns | ns | ns |
| Velocity |  |  |  |  | ns | ns | ns | ns | ns | 0.58 | -0.98 | ns | ns | ns |
| Water temp |  |  |  |  |  | 0.49 | ns | ns | ns | 0.31 | ns | ns | 0.43 | -0.94 |
| Conductivity |  |  |  |  |  |  | ns | ns | ns | 0.89 | -0.56 | -0.20 | 0.20 | -0.71 |
| NH_4_^+^ |  |  |  |  |  |  |  | 0.44 | 0.23 | ns | -0.69 | ns | ns | ns |
| PO_4_^3-^ |  |  |  |  |  |  |  |  | ns | ns | ns | 0.70 | 0.70 | ns |
| NO_3_^-^ |  |  |  |  |  |  |  |  |  | ns | ns | ns | -0.83 | ns |
| Si |  |  |  |  |  |  |  |  |  |  | -0.56 | ns | ns | -0.60 |
| Julian day |  |  |  |  |  |  |  |  |  |  |  | ns | ns | 0.25 |
| Catchment NDVI |  |  |  |  |  |  |  |  |  |  |  |  | 0.89 | ns |
| Catchment area |  |  |  |  |  |  |  |  |  |  |  |  |  | -0.37 |
| catchment slope |  |  |  |  |  |  |  |  |  |  |  |  |  |  |
